# Behavioral Health and the Comprehensive Primary Care (CPC) Initiative: findings from the 2014 CPC behavioral health survey

**DOI:** 10.1186/s12913-017-2562-z

**Published:** 2017-08-29

**Authors:** Kara Zivin, Benjamin F. Miller, Bruce Finke, Asaf Bitton, Perry Payne, Edith C. Stowe, Ashok Reddy, Timothy J. Day, Pauline Lapin, Janel L. Jin, Laura L. Sessums

**Affiliations:** 1 Department of Veterans Affairs, Center for Clinical Management Research, 2800 Plymouth Road, Ann Arbor, MI 48109 USA; 2 Department of Psychiatry, University of Michigan Medical School, 2800 Plymouth Road, Ann Arbor, MI 48109 USA; 3 Department of Family Medicine, University of Colorado School of Medicine, 12631 East 17th Ave, Aurora, CO 80045 USA; 4 Center for Medicare and Medicaid Innovation, Centers for Medicare and Medicaid Services, 7500 Security Boulevard, Baltimore, MD 21244 USA; 5 Nashville Area Indian Health Service, 711 Stewarts Ferry Pike, Nashville, TN 37214 USA; 6 Brigham and Women’s Hospital, Harvard Medical School, 75 Francis Street, Boston, MA 02115 USA; 7 Department of Medicine, University of Washington, 325 Ninth Ave, Campus Box 359780, Seattle, WA 98104 USA

**Keywords:** Behavioral health integration, Primary care, Patient centered medical homes, Costs

## Abstract

**Background:**

Incorporating behavioral health care into patient centered medical homes is critical for improving patient health and care quality while reducing costs. Despite documented effectiveness of behavioral health integration (BHI) in primary care settings, implementation is limited outside of large health systems. We conducted a survey of BHI in primary care practices participating in the Comprehensive Primary Care (CPC) initiative, a four-year multi-payer initiative of the Centers for Medicare and Medicaid Services (CMS). We sought to explore associations between practice characteristics and the extent of BHI to illuminate possible factors influencing successful implementation.

**Method:**

We fielded a survey that addressed six substantive domains (integrated space, training, access, communication and coordination, treatment planning, and available resources) and five behavioral health conditions (depression, anxiety, pain, alcohol use disorder, and cognitive function). Descriptive statistics compared BHI survey respondents to all CPC practices, documented the availability of behavioral health providers, and primary care and behavioral health provider communication. Bivariate relationships compared provider and practice characteristics and domain scores.

**Results:**

One hundred sixty-one of 188 eligible primary care practices completed the survey (86% response rate). Scores indicated basic to good baseline implementation of BHI in all domains, with lowest scores on communication and coordination and highest scores for depression. Higher scores were associated with: having any behavioral health provider, multispecialty practice, patient-centered medical home designation, and having any communication between behavioral health and primary care providers.

**Conclusions:**

This study provides useful data on opportunities and challenges of scaling BHI integration linked to primary care transformation. Payment reform models such as CPC can assist in BHI promotion and development.

**Electronic supplementary material:**

The online version of this article (10.1186/s12913-017-2562-z) contains supplementary material, which is available to authorized users.

## Background

Primary care plays a critical role in health care and payment reform redesign. One of the most promising new care models is the Patient-Centered Medical Home (PCMH), which aims to treat the whole person, not only particular conditions or body parts, in a timely, coordinated, and comprehensive manner [1–5]. There has been increasing awareness that despite historically based separation in clinical training, treatment locations, insurance coverage, and billing practices in the United States, it is virtually impossible to separate behavioral health care needs from primary care [6]. Behavioral health conditions, which encompass mental health and substance abuse disorders, are usually identified and treated in primary care settings, [7, 8] a setting in which patients often prefer to receive behavioral health care [9]. Many consider it essential to have behavioral health care providers in primary care settings to promote health behavior change, treatment adherence, develop comprehensive treatment plans, and improve outcomes [7, 10, 11]. Given the high prevalence of both behavioral health conditions, and psychiatric comorbidities among individuals with chronic medical conditions, PCMHs that address behavioral health care needs could improve patient health and care quality and reduce costs [11–19].

Evidence-based approaches to addressing behavioral health needs in primary care often focus on integrating behavioral health providers with the primary care team [20, 21]. Behavioral health integration (BHI) involves care conducted with primary care and behavioral health providers working together with patients and families to provide systematic, patient-centered care [22, 23]. Examples include assisting a patient with health behavior change or performing an evidence-based intervention for depression. Multiple studies have demonstrated effectiveness of team-based approaches to managing behavioral health conditions in primary care [24] and addressing behavioral health and physical health needs simultaneously [25, 26].

In 2007, the American Academy of Family Physicians, American Academy of Pediatrics, American College of Physicians, American Osteopathic Association developed initial PCMH principles, and in 2014, they developed joint principles for BHI in PCMHs [13]. Although numerous randomized controlled trials have found that BHI improves care processes and outcomes, [27] and in spite of recommendations to incorporate BHI into PCMHs, widespread adoption of BHI has not occurred, [7] particularly outside of large integrated health systems (e.g., Department of Veterans Affairs, which mandated integrated care in 2008) [28].

Increasingly, primary care practices are conducting BHI [29]. However, several barriers exist to integrating behavioral health providers into primary care [30], including payment [10, 31, 32]. Primary care practices interested in BHI typically have fragmented payment models, making sustainable funding difficult. However, despite historical differences leading to separate payments for behavioral health and medical services, novel payment models exist aiming to better support primary care BHI.

A key challenge to measuring BHI implementation in primary care practices is that it is difficult to ascertain whether, how, and to what extent practices have adopted BHI. Most studies that evaluate any type of health care use employ administrative data to document the presence or absence of a particular diagnosis or treatment. However, being able to discern whether practices have co-located providers or whether primary care physicians and behavioral health providers communicate with one another is virtually unknowable from these data sources. Therefore, using surveys to learn more about whether and how BHI is operationalized within a given practice provides valuable and typically unavailable information.

This survey provides a benchmark to compare future assessments of BHI, both with and outside of CPC, including those that will become feasible with the implementation of Medicare payment for BHI (i.e., four new codes created for the 2017 Medicare Physician Fee Schedule to allow payment to health care providers for furnishing BHI services) in 2017 [33]. Further, our study identifies key associations between practice characteristics and the extent of BHI implementation, which will allow us to illuminate possible factors that facilitate success of implementing BHI.

### The Comprehensive Primary Care (CPC) Initiative

CPC is a four-year multi-payer initiative (2012-2016) of the Centers for Medicare and Medicaid Services (CMS) in seven regions across the US designed to strengthen primary care [34]. CMS and other payers pay nearly 500 participating primary care practices with approximately 2,700,000 patients quarterly population-based care management fees in addition to usual fee for service payments, and provide shared savings opportunities to support providing a core set of comprehensive primary care functions. In 2013 (the first year of the initiative), CPC practices received sizable enhanced payments from CMS and other participating payers: CPC care management fees for the median practice were about $227,849 ($70,045 per clinician or $137 per attributed patient), equivalent to 19% of 2012 total practice revenue for the median practice [34]. Practices were required to provide risk-stratified care management to patients at highest risk for poor outcomes and preventable harm. Additional details on CPC’s design are presented elsewhere [35, 36].

In CPC’s second year, practices were required to engage in at least one of three advanced primary care strategies: 1) BHI, 2) comprehensive medication management, and 3) self-management support. Practices choosing to focus on BHI reported quarterly about their progress towards implementing BHI, and these questions provided implicit guidance to practices on BHI. Questions included: 1) who provides behavioral health services and what services they provide; 2) how practices identify and treat patients with behavioral health needs; 3) which tools practices used to assess patients behavioral health needs and monitor care; 4) what evidence-based treatments practices provided; 5) whether and how practices engaged in systematic case review and consultation; 6) how practices were building capacity for behavioral health; 7) how practices were currently tracking patients receiving behavioral health care; and 8) how practices measure their progress towards BHI.

The 2014 CPC Behavioral Health Survey was designed to assess progress towards implementing BHI in practices that chose BHI as their advanced primary care strategy. CPC programmatic requirements provided a framework for BHI, including multiple regional and national learning sessions. There were three overarching components of this framework. Each participating practice needed to: 1) be able to identify and meet patient behavioral health needs through co-management or coordinated referral; 2) use standardized assessment measures, evidence-based treatment, patient and family engagement strategies including shared decision making, and conduct appropriate follow-up and treatment adjustment as needed; and 3) measure the impact of BHI and adapt as needed to improve outcomes.

Despite this framework, explicitly absent was a prescriptive approach for how practices needed to conduct BHI. Practices were not required to have behavioral health providers co-located within their facilities, as BHI was only one component of a much larger overall PCMH model test. It would not have been feasible to select practices to participate in CPC (not all of which focused on BHI) on the basis of whether a behavioral health provider was available onsite. However, given CPC’s flexibility of approaches to potential BHI implementation, it is possible that practices interpreted the definition of BHI, and thus the implementation of BHI, in different ways. Findings from this survey will provide a useful benchmark to compare future developments in BHI, both within CPC practices, and among other practices seeking to implement and further develop BHI. This study is unique in that it assesses BHI implementation in practices undergoing care redesign supported by payment reform across seven regions in primary care practice settings. It explores associations between practice characteristics and the extent of BHI to identify possible factors influencing successful implementation.

## Methods

### Participants

All CPC practices that elected to focus on BHI or that had a behavioral health provider on staff were eligible for survey participation (*N*=188 out of 483 total CPC practices). We emailed the surveys to practice CPC point of contact, requesting that each eligible practice complete one survey. Survey instructions encouraged respondents to discuss the survey questions prior to answering them with clinicians, other clinical staff, and non-clinical staff.

### Data sources and practice characteristics

In addition to the BHI survey, we linked survey data to CPC application data, which data included information collected in 2012 from all practices that applied to become part of CPC: whether the practice was part of a multispecialty practice, practice ownership status (hospital, physician, or government/other owned), whether the practice qualified for National Center for Quality Assurance PCMH designation, [37] metropolitan area (yes, no), patient mix (proportion of African American patients in the practice), and patients per full time equivalent (FTE) provider.

### Survey measures

CMS constructed the 2014 Behavioral Health Integration Survey (BHI Survey; Additional file 1), modeling it after the Patient-Centered Medical Home Assessment (PCMH-A) tool [38] and its precursor, the Assessment of Chronic Illness Care (ACIC) [39]. PCMH-A defines practice characteristics and behaviors that comprise a PCMH, including describing the trajectory to full implementation, and it is used in the independent evaluation of CPC [34]. Similar to these survey tools, the BHI survey measures implementation along a spectrum including limited, basic, good, and full implementation.

Similar to PCMH-A, we sought to define practice characteristics that comprise BHI, to assist practices in characterizing progress towards and identifying opportunities for improving BHI implementation [38]. The scored portion of the BHI survey included 23 items in 11 domains, with 6 substantive areas (integrated space, training, access, communication and coordination, treatment planning, and available resources) and 5 disorders (depression, anxiety, pain, alcohol use disorder, and cognitive function). We identified one to four actionable changes for each domain. Questions were scored from 1 to 4, with higher scores indicating greater implementation. Scores indicated limited, basic, good, and full implementation respectively. Domain scores were purposely not aggregated so that practices could identify separate areas of strength and opportunities for improvement.

Additional survey questions that were not scored asked practices to document the number and type of behavioral health providers (e.g., psychiatrists, social workers, psychologists, marriage and family therapists, psychiatric nurse practitioners, other) available in their practice, and years of experience in outpatient treatment, length of time affiliated with CPC, and proportion of time involved with CPC. A final question asked about communication approaches between primary care providers (PCPs) and behavioral health providers (e.g., conversations in person or by telephone, notes from either PCPs or behavioral health providers, or no communication).

This survey focused on baseline implementation efforts and does not address outcomes of BHI in CPC practices. Mathematica Policy Research conducted an independent evaluation of CPC, with findings reported elsewhere [34, 40, 41].

### Survey fielding

CPC practices elected BHI or another advanced primary care strategy in their regular reporting to CMS for the first quarter 2014 due in April 2014. In June 2014, all CPC practices were notified by electronic communication about the survey. The survey was open between July and September 2014, and eligible practices received an invitation to participate that included an online link and instructions for completing it. Since BHI survey data were collected as part of quality improvement rather than a formal research project, practices were not required nor given incentives to return surveys. Participation was strongly encouraged, and practices received up to five reminders to complete the survey. Practices that initiated a survey but did not complete it (i.e., had any missing data on scored items), were asked to redo the survey. All practices that initiated a survey ultimately completed the survey.

### Analysis plan

We generated descriptive statistics to compare BHI survey respondents to all CPC practices, and conducted χ^2^ tests to identify any significant differences between respondents and non-respondents on practice characteristics. We generated descriptive statistics for responding practices regarding availability of behavioral health providers and PCP/behavioral health provider communication. We calculated 11 domain scores, and examined bivariate relationships using χ^2^ tests between provider and practice characteristics and domain scores. All analyses were conducted using SAS software Version 9.3 (SAS Inc., Cary, NC). A statistical significance level of *p*<0.05 was used for all analyses.

## Results

We received 161 survey responses (86% response rate). Table 1 presents practice characteristics of BHI survey respondents and CPC practices overall. Oregon was disproportionately represented among BHI respondents relative to the number of practices from Oregon in CPC. Similar to CPC overall, the preponderance of BHI respondents were from single specialty practices and urban areas, and a substantial minority had achieved PCMH status at the time they applied to join CPC.

**Table 1:** Characteristics of the 161 practices that responded to the 2014 Behavioral Health Integration Survey and characteristics of all Comprehensive Primary Care (CPC) initiative practices^a^

	BHI survey respondents (*N*=161)		All practices (*N*=483)	
	N or mean	% or SE	N or mean	% or SE
Region				
Arkansas	15	9.3	63	13.0
Colorado	34	21.1	73	15.1
New Jersey	24	14.9	67	13.9
New York	5	3.1	74	15.3
Ohio	6	3.7	75	15.5
Oklahoma	27	16.8	64	13.3
Oregon	50	31.1	67	13.9
Multispecialty practice	24	14.9	59	12.2
Ownership				
Hospital owned	60	37.3	214	44.3
Physician owned	92	57.1	258	53.4
Government or other owned	8	5.0	9	1.9
PCMH designation	67	41.6	176	36.4
Metropolitan area	126	78.3	391	81.0
Patient mix: % African American (mean, SE)	4.2	7.0	4.9	7.7
Patients per FTE physician in practice (mean, SE)	1,605	1,342	1,790	3,124

^a^N and % unless otherwise noted

We found expected differences between survey respondents and survey non-respondents. There were regional differences across the seven regions in practices that did and did not respond to the survey (χ^2^ = 17.47, *p*<0.01) and BHI respondents were more likely than non-respondents to be from a multiple specialty practice (χ^2^ =161.00, *p*<0.0001).

Based on survey data, a majority of practices had behavioral health provider(s) in their practice (Table 2). Of provider types assessed, psychologists and social workers were the most commonly available; only a small percentage of practices reported having a psychiatrist. A small proportion of practices that did not have a behavioral health provider indicated that they were working on getting one. Of practices that reported having behavioral health providers, practices reported having highly experienced behavioral health providers (a majority of whom had more than five years of experience), many of whom had been involved with the practice for at least 12 months, and with behavioral health professionals spending at least half of their time with the CPC practice. In response to a question that asked practices to identify forms of communication (e.g., in person, by telephone, PCP progress notes, psychotherapy notes from behavioral health provider) between behavioral health providers and PCPs, the vast majority of all practices reported having some form of communication.

**Table 2:** Inclusion and characteristics of behavioral health care providers in CPC practices that responded to the 2014 Behavioral Health Integration Survey

Practice characteristic	N	%
Behavioral health providers (N=161)*		
Any behavioral health clinician onsite	86	53.4%
Psychiatrist	11	6.8%
Psychologist	38	23.6%
Social worker	40	24.8%
Marriage and family therapist	8	5.0%
Psychiatric nurse practitioner	4	2.5%
Other behavioral health provider	34	21.1%
No behavioral health provider	75	46.6%
Working on getting a behavioral health provider	12	7.5%
Any PCP and BHP communication (in person, telephone, notes) (N=161)	134	83.2%
Years of direct care in an outpatient setting (N=102, 59 missing)		
<1 year	29	28.4%
1-5 years	27	26.5%
>5 years	46	45.1%
How long has provider been involved with CPC practice (N=100, 61 missing)		
<6 months	35	35.0%
6-12 months	19	19.0%
>12 months	46	46.0%
Proportion of time spent with CPC practice (N=99, 62 missing)		
Ad hoc consultant	6	6.1%
<25%	24	24.2%
25-50%	14	14.1%
>50%	55	55.6%

*Practices could have more than one type of provider. Other providers noted included licensed professional counselor (LPC), pharmacist, unspecified MD/PhD/MA, alcohol and drug counselor, registered nurse, unspecified mental health professional

Respondent scores for all survey domains ranged from 2.02 (sd=0.91; communication and coordination) to 3.24 (sd=0.69; depression), indicating that most practices had basic to good (but not full) support for BHI in each domain assessed (Tables 3 and 4) [39]. Practice characteristics associated with higher domain scores included: multispecialty practice, PCMH designation, availability of any behavioral health provider, and PCP/behavioral health provider communication (Tables 3 and 4). Scores varied significantly by region. Ownership status, patients/FTE, and metropolitan area had limited relationship to domain scores. Within individual domains, practice characteristics had a variable impact on domain scores. Many substantive domains were sensitive to practice characteristics. Specifically, access, communication and coordination, and treatment planning were significantly positively associated with being a multispecialty practice, having PCMH designation, and having any behavioral health provider on staff. Conversely, disorder-related domain scores were not significantly influenced by most practice characteristics, other than differences by region or availability of any behavioral health provider.

**Table 3:** Bivariate relationships between practice characteristics and substantive domain scores in the 2014 Behavioral Health Integration Survey

	Integrated space	Training	Access	Communication and coordination	Treatment planning	Available resources
Mean domain score	2.385	2.366	2.646	2.019	2.358	2.379
Standard deviation	1.240	0.642	0.845	0.915	0.798	0.955
F-tests						
Region	6.716***	2.164*	5.786***	6.244***	6.829***	4.139**
Multispecialty practice	5.321*	0.005	12.401**	6.099*	6.718*	12.815***
Practice ownership						
Hospital owned	35.026***	0	0.389	2.247	1.612	0.807
Physician owned	25.187***	0.101	0.416	1.583	0.445	0.412
Government or other owned	0.811	0.275	0.325	0.066	1.466	0.153
Patient centered medical home	2.93	28.377***	7.531**	4.304*	8.005**	7.096**
Metropolitan area	0.005	2.711	0.773	0.201	0.694	0.022
Behavioral health providers in the practice						
Psychiatrist	1.444	2.604	4.050*	0.910	6.833*	0.858
Social worker	32.856***	9.052**	35.905***	35.967***	14.749***	19.412***
Psychologist	27.316***	0.788	25.600***	39.411***	18.616***	19.689***
Marriage and family therapist	3.034	2.405	5.378*	8.910**	7.910**	3.620
Psychiatric nurse practitioner	5.095*	9.801**	5.663*	4.832*	6.149*	0.618
Other behavioral health provider	9.103**	4.357*	15.571***	5.404*	9.042**	2.086
Any type of behavioral health provider	95.773***	15.844***	81.823***	85.982***	42.850***	27.631***
No provider available but working on getting one	3.232	5.761*	19.406***	8.573**	15.363***	27.878***
Any communication between PCP and BHP	0.195	12.92***	25.809***	30.476***	24.751***	38.31***
Mean number of African American patients	6.721*	2.840	5.536*	3.593	3.067	0.006
Mean number of full time equivalent (FTE) providers	2.175	0.090	2.525	3.606	5.873*	2.662

*p<0.05; **p<0.01, ***p<0.001; domain scores range from 1-4; all F-tests have 1 degree of freedom (df) except region, which has 6 df

**Table 4:** Bivariate relationships between practice characteristics and condition domain scores in the 2014 Behavioral Health Integration Survey

	Depression	Anxiety	Pain	Alcohol use disorder	Cognitive function
Mean domain score	3.242	2.832	2.891	2.634	2.689
Standard deviation	0.689	0.900	0.864	0.860	0.810
F-tests					
Region	4.852***	5.306***	10.045***	6.753***	3.814**
Multispecialty practice	0.491	0.063	0.052	0.717	0.482
Practice ownership					
Hospital owned	2.407	2.083	0.010	0.035	0.001
Physician owned	0.947	1.298	0.415	0.784	0.254
Government or other owned	1.826	1.824	2.668	3.547	1.271
Patient centered medical home	8.475**	8.173**	6.744*	3.196	3.061
Metropolitan area	1.948	0.674	0.533	1.403	0.022
Behavioral health providers in the practice					
Psychiatrist	3.059	0.662	7.028**	4.113*	0.026
Social worker	3.293	2.151	5.919*	16.236***	0.009
Psychologist	6.475*	4.233*	9.723**	6.864*	0.277
Marriage and family therapist	3.568	0.004	0.003	0.436	1.833
Psychiatric nurse practitioner	3.509	4.359*	4.134*	5.607*	9.24**
Other behavioral health provider	4.222*	1.019	0.509	2.463	0.137
Any type of behavioral health provider	12.080**	4.103*	22.504***	19.993***	2.589
No provider available but working on getting one	35.436***	13.170***	30.950***	7.312**	6.514*
Any communication between PCP and BHP	16.113***	4.524*	24.927***	10.101**	5.165*
Mean number of African American patients	0.293	0.260	0.010	3.933*	1.263
Mean number of full time equivalent (FTE) providers	1.099	0.451	0.147	0.348	2.055

*p<0.05; **p<0.01, ***p<0.001; domain scores range from 1-4; all F-tests have 1 degree of freedom (df) except region, which has 6 df

Answers to individual questions within a given domain illustrate variation possible within that domain (see Additional file 2). For example, the mean score for the integrated space domain question was 2.39 (sd=1.24; Table 3). Within that domain, the individual item question found that 41% of practices had entirely separate space, with the remaining 59% of practices having some level of integrated space, including 24% with fully integrated space (i.e., clinic space shared between behavioral health providers and PCPs). In the training domain, most practices reported that PCPs had been trained in principles of behavioral health care, with a mixture of those who reported feeling comfortable handling these issues and others not. In contrast, other clinical professionals and non-clinic staff in most practices did not address behavioral health issues or play a very limited role. In the access domain focused on scheduling, the preponderance of practices reported PCPs’ ability to accommodate addressing behavioral health issues, but a much more limited number of practices had the ability for behavioral health providers to accommodate addressing behavioral health issues. In the communication and coordination domain, respondents indicated that PCPs and behavioral health providers engaged in limited communication and did not regularly meet to review cases. In the treatment planning domain, shared care plans between PCP and behavioral health providers were rarely recorded and shared electronic health records were not routinely available. Many practices were able to link patients to community resources and follow up with patients regarding their behavioral health needs. In the resource domain, most practices reported not having readily available resources for patients’ behavioral health needs, such as staff and time.

Findings from disease-specific domains revealed high levels of disease screening (depression, anxiety, pain, alcohol use disorder, and cognitive function; Additional file 2). However, practices had treatment targets and consistent outcome monitoring for depression, anxiety, and pain; practices did not regularly provide care for alcohol use disorders or address cognitive disorders, nor did they typically have treatment targets for these disorders.

## Discussion

This paper provides a unique assessment of the real-world baseline status of BHI implementation in a multi-state primary care redesign initiative. We found that being part of a multispecialty practice, having a PCMH designation, having any behavioral health provider (especially a psychologist or social worker), and any communication between behavioral health providers and PCPs were each associated with significantly higher BHI domain and disease area scores. Conversely, practice ownership, metropolitan location, proportion of African American patients, and number of patients per FTE provider had almost no discernible impact on scores. We are unaware of any other survey that assesses BHI across practices, let alone one that includes this many primary care practices and patients, and that documents a broad range of experiences at the initiation or expansion BHI, particularly within the context of a primary care redesign effort. BHI is typically inferred from administrative data, and this survey provides key findings not available from those data sources.

Our study had several strengths including its breadth, depth, and response rate. We achieved a high response rate to this survey because practices were accustomed to regular reporting requirements as part of CPC participation, in addition to the multiple reminders they received to complete the survey. Findings also identified strengths and opportunities for improvement in BHI. Bringing together two historically disparate clinical treatment disciplines and cultures can be challenging and rewarding, and consistent with other findings, behavioral health providers were co-located in practices only roughly half of the time, with a very limited number of practices having any psychiatrists available [29]. Alternatively, most practices (83%) indicated that they had some form of communication with behavioral health providers, and nearly half (46%) had a behavioral health provider who had been involved with the practice for at least one year. Although regional differences were not a focus of this paper, regions that had initiated BHI prior to CPC were both more likely to be represented in the survey and were more likely to have higher domain scores (e.g., Oregon; data not shown).

Large national model tests programs such as CPC are investing substantial financial and educational resources in assisting practices to move from encounter-based, fee-for-service patient care towards a team-based, patient-centered approach with population-based payments. In the future, an increasing proportion of Medicare payments will be tied to quality or value, [42] which will likely continue to support BHI goals. As an essential element of primary care, inclusion of behavioral health holds great promise. As the survey indicates, however, primary practices have multiple opportunities to deepen their BHI work.

Though no causality can be implied from survey results, primary practices with experience in expanding the medical neighborhood, working with other medical specialties (as in multispecialty practices) and developing strong communication between a provider and a specialist, had a higher degree of BHI implementation. For policymakers and practices integrating behavioral health, these insights provide new and useful data on opportunities and challenges of BHI integration linked to primary care transformation.

Survey limitations include that it was cross-sectional given at the start of BHI implementation among CPC practices; it does not address how BHI implementation changes over time. We did not formally evaluate survey psychometric properties (such as inter-rater reliability) nor conduct a validation study prior to fielding this quality improvement tool. We did not link survey responses to staff or patient satisfaction or cost data, although the overall independent CPC evaluation could do that work. Domain scores were likely substantially higher than typical PC practices, as included practices chose to participate in BHI and CPC, therefore, may not be representative of typical primary care practices. It is also unknown whether findings could be replicated in other practices with less financial and technical support, though increasing numbers of practice are being paid differently for primary care services [43]. Finally, we are not certain who within each practice completed each survey, and whether administrators included front line clinicians when completing the survey.

A final concern is the lack of a consistent definition of BHI, which makes assessment of progress towards BHI challenging. As others have noted, the field needs a “lexicon” that can consistently explain what BHI is and is not [23]. Tools such as the Agency for Healthcare Research and Quality (AHRQ) lexicon have been created to help further elucidate what is and what is not BHI [44]. Implementing such tools would allow for a clearer distinction that helps with better identification and implementation of BHI. Since many practices do not have onsite behavioral health providers, future research using clearer criteria could better delineate between practices with fully integrated clinical workflows and those that provide high degrees of coordination and co-management between primary care and behavioral health external to the practice.

## Conclusions

This study found a few key factors that influenced BHI, some of which may be more easily attainable for practices than others. It may be easier to develop collaboration with a psychologist or social worker than a psychiatrist. Focusing specifically on communication and/or securing access to any type of behavioral health provider may assist practices towards successful BHI implementation, even in the context of significant variation in practice size, location, and patient demographic characteristics.

Innovative multi-payer initiatives such as CPC hold great promise for catalyzing primary care to more effectively address needs of patients with behavioral health needs. To continue to promulgate BHI, there must be adequate practice transformation supports that allow for variability in BHI implementation while maintaining consistency with an overall definitional framework [22, 23]. Multi-payer alignment regarding BHI should be encouraged and continue. Future studies should examine the impact of new payment models and billing practices on BHI to help achieve the value added proposition of BHI, and should assess BHI-related outcomes.

## Additional files

Additional file 1: 2014 Behavioral Health Integration Survey. (PDF 432 kb)

Additional file 2: 2014 Behavioral Health Integration Survey item level responses. Comprehensive Primary Care application data and the Behavioral Health Integration survey data. (PDF 72 kb)

## Abbreviations

ACIC: Assessment of Chronic Illness Care
BHI: Behavioral health integration
BHI Survey: Behavioral Health Integration Survey
CPC: Comprehensive Primary Care
CMS: Centers for Medicare and Medicaid Services
FTE: Full time equivalent
PCMH: Patient-Centered Medical Home
PCMH-A: Patient-Centered Medical Home Assessment
