# Supplementary material for: Behavioral Health and the Comprehensive Primary Care (CPC) Initiative: findings from the 2014 CPC behavioral health survey
Source: BMC Health Serv Res. 2017 Aug 29;17:612. doi: 10.1186/s12913-017-2562-z (PMC5576039; doi:10.1186/s12913-017-2562-z)
Supplement: Supplementary file 1 — 2014 Behavioral Health Integration Survey. (PDF 432 kb) [file 12913_2017_2562_MOESM1_ESM.pdf]

2014 CPC Behavioral Health Integration Survey

CPC Practice ID *

This survey is designed to help practices move toward delivering integrated behavioral health care. The results can be used to identify progress as well as help your team identify areas for improvement.

We encourage you to discuss the survey questions prior to answering them with clinicians, other clinical staff, and non-clinical staff. Please answer each question from the perspective of one practice.

How many of each of the following Behavioral Health Providers (BHPs; in terms of full time equivalents [FTEs]), if any, work at your main practice location?

	0	1	2 to 5	6 to 10	>10
**Psychiatrists**	○	○	○	○	○
**Social Workers**	○	○	○	○	○
**Psychologists**	○	○	○	○	○
**Marriage and Family Therapists (MFT)**	○	○	○	○	○
**Psychiatric Nurse Practitioners**	○	○	○	○	○
**Other**	○	○	○	○	○

For each of the providers listed above, answer the following questions (for those that do not apply to your setting based on what you reported above, you can leave them blank):

Psychiatrist 1

	<1 year	1 year	1-5 years	>5 years
**Years of direct patient care in outpatient medical setting**	○	○	○	○

Psychiatrist 1

	<6 months	6-12 months	>12 months
**How long has provider been involved with CPC practice**	○	○	○

Psychiatrist 1

	Ad hoc consultant	<25%	25-50%	>50%
**Proportion of time involved with CPC practice**	○	○	○	○

Psychiatrist 2

	<1 year	1 year	1-5 years	>5 years
**Years of direct patient care in outpatient medical setting**	○	○	○	○

Psychiatrist 2

	<6 months	6-12 months	>12 months
**How long has provider been involved with CPC practice**	○	○	○

Psychiatrist 2

	Ad hoc consultant	<25%	25-50%	>50%
**Proportion of time involved with CPC practice**	○	○	○	○

Social Worker 1

	<1 year	1 year	1-5 years	>5 years
**Years of direct patient care in outpatient medical setting**	○	○	○	○

Social Worker 1

	<6 months	6-12 months	>12 months
**How long has provider been involved with CPC practice**	○	○	○

Social Worker 1

	Ad hoc consultant	<25%	25-50%	>50%
**Proportion of time involved with CPC practice**	○	○	○	○

Social Worker 2

	<1 year	1 year	1-5 years	>5 years
**Years of direct patient care in outpatient medical setting**	○	○	○	○

Social Worker 2

	<6 months	6-12 months	>12 months
**How long has provider been involved with CPC practice**	○	○	○

Social Worker 2

	Ad hoc consultant	<25%	25-50%	>50%
**Proportion of time involved with CPC practice**	○	○	○	○

Psychologist 1

	<1 year	1 year	1-5 years	>5 years
**Years of direct patient care in outpatient medical setting**	○	○	○	○

Psychologist 1

	<6 months	6-12 months	>12 months
**How long has provider been involved with CPC practice**	○	○	○

Psychologist 1

	Ad hoc consultant	<25%	25-50%	>50%
**Proportion of time involved with CPC practice**	○	○	○	○

Psychologist 2

	<1 year	1 year	1-5 years	>5 years
**Years of direct patient care in outpatient medical setting**	○	○	○	○

Psychologist 2

	<6 months	6-12 months	>12 months
**How long has provider been involved with CPC practice**	○	○	○

Psychologist 2

	Ad hoc consultant	<25%	25-50%	>50%
**Proportion of time involved with CPC practice**	○	○	○	○

Marriage and Family Therapist 1

	<1 year	1 year	1-5 years	>5 years
**Years of direct patient care in outpatient medical setting**	○	○	○	○

Marriage and Family Therapist 1

	<6 months	6-12 months	>12 months
**How long has provider been involved with CPC practice**	○	○	○

Marriage and Family Therapist 1

	Ad hoc consultant	<25%	25-50%	>50%
**Proportion of time involved with CPC practice**	○	○	○	○

Marriage and Family Therapist 2

	<1 year	1 year	1-5 years	>5 years
**Years of direct patient care in outpatient medical setting**	○	○	○	○

Marriage and Family Therapist 2

	<6 months	6-12 months	>12 months
**How long has provider been involved with CPC practice**	○	○	○

Marriage and Family Therapist 2

	Ad hoc consultant	<25%	25-50%	>50%
**Proportion of time involved with CPC practice**	○	○	○	○

Psychiatric Nurse Practitioners 1

	<1 year	1 year	1-5 years	>5 years
**Years of direct patient care in outpatient medical setting**	○	○	○	○

Psychiatric Nurse Practitioners 1

	<6 months	6-12 months	>12 months
**How long has provider been involved with CPC practice**	○	○	○

Psychiatric Nurse Practitioners 1

	Ad hoc consultant	<25%	25-50%	>50%
**Proportion of time involved with CPC practice**				

Psychiatric Nurse Practitioners 2

	<1 year	1 year	1-5 years	>5 years
**Years of direct patient care in outpatient medical setting**				

Psychiatric Nurse Practitioners 2

	<6 months	6-12 months	>12 months
**How long has provider been involved with CPC practice**			

Psychiatric Nurse Practitioners 2

	Ad hoc consultant	<25%	25-50%	>50%
**Proportion of time involved with CPC practice**				

Other providers (please specify)

Other 1

	<1 year	1 year	1-5 years	>5 years
**Years of direct patient care in outpatient medical setting**				

Other 1

	<6 months	6-12 months	>12 months
**How long has provider been involved with CPC practice**			

Other 1

	Ad hoc consultant	<25%	25-50%	>50%
**Proportion of time involved with CPC practice**				

Other 2

	<1 year	1 year	1-5 years	>5 years
**Years of direct patient care in outpatient medical setting**				

Other 2

	<6 months	6-12 months	>12 months
**How long has provider been involved with CPC practice**			

Other 2

	Ad hoc consultant	<25%	25-50%	>50%
**Proportion of time involved with CPC practice**				

Please list any additional providers and their years of direct patient care in outpatient medical settings, how long the provider has been involved with CPC practice, and the proportion of time involved with CPC practice

2014 CPC Behavioral Health Integration Survey

For each row, click the point value that best describes the level of care that currently exists in your practice. The rows in this form present key aspects of integrated behavioral health care. Each aspect is divided into levels showing various stages in development toward behavioral health integration. The stages are represented by points that range from 1 to 4. The higher point values indicate that the actions described in that box are more fully implemented.

Integrated space

	Entirely separate space	Mostly separate space: PCPs and BHPs spend little time with each other practicing in same clinic space. Patient has to see providers in at least two buildings	Co-located space: Behavioral health and medical clinicians in different parts of the same building, spending some but not all their time in same medical clinic space. Patient typically has to move from primary care to behavioral health space	Fully shared space: Behavioral health and medical clinicians share the same provider rooms, spending all or most of their time seeing patients in that shared space. Typically, the clinicians see the patient in same exam room.
**Type of spatial arrangement employed**	1	2	3	4

Training

	Do not address behavioral health care issues	Play a limited role in providing behavioral health care	Have been trained in principles of behavioral health care but largely rely on others to address these issues	Have been trained in principles of behavioral health care and feel comfortable handling the majority of routine behavioral health needs
**PCPs**	1	2	3	4

	Do not address behavioral health care issues	Play a limited role in providing behavioral health care	Have been trained in principles of behavioral health care but largely rely on others to address these issues	Have been trained in principles of behavioral health care and feel comfortable handling the majority of routine behavioral health needs as allowed by their license
**Other clinical staff**	1	2	3	4

	Do not address behavioral health care issues	Are primarily tasked with managing patient flow and triage regarding behavioral health needs	Provide some services such as assessment or screening for behavioral health conditions	Perform key service roles that match their abilities and position
**Non-clinical staff**	1	2	3	4

Access

	Are not available at the practice	Can be scheduled by the practice with some flexibility in scheduling different visit lengths	Provide flexibility and include capacity for same day visits	Are flexible and can accommodate customized visit lengths, same day visits, schedule follow-up, and multiple provider visits
**Appointments with PCPs to address behavioral health needs**	1	2	3	4

	Are not	Can be	Provide	Are flexible and

	available at the practice	scheduled by the practice with some flexibility in scheduling different visit lengths	flexibility and include capacity for same day visits	can accommodate customized visit lengths, same day visits, schedule follow-up, and multiple provider visits
**Appointments with BHPs to address behavioral health needs**	1	2	3	4

Communication and coordination

	Do not regularly communicate; BHPs are not part of the practice	Engage in periodic information exchanges with minimally shared care plans or workflows	Engage in regular communication and coordination, usually via separate systems and workflows, but with care plans coordinated to a significant extent	Have regular communication facilitated and/or clinical workflows that ensure effective communication and coordination
**PCPs and BHPs**	1	2	3	4

	PCPs and BHPs do not meet regularly to review cases	PCPs and BHPs meet periodically to conduct caseload review on patients who are not improving	PCPs and BHPs meet routinely to conduct caseload review on patients who are not improving	PCPs and BHPs meet routinely to conduct caseload review on patients who are not improving; BHPs provide specific recommendations for treatment changes or referrals, and provide psychiatric assessment when indicated

**Case review**	1	2	3	4

What approaches do PCPs and BHPs use to communicate about patient needs (check all that apply):

- No feedback
- In person conversations
- Telephone conversations
- Progress notes from PCP
- Psychotherapy notes from BHP
- Not applicable

Treatment planning

	Are not routinely developed or recorded	Are developed and recorded but reflect PCP and BHPs' separate priorities only	Are developed collaboratively with PCPs and BHPs and include behavioral self-management and clinical goals, but they are not routinely used to guide subsequent care	Are developed collaboratively with PCPs and BHPs as well as patients and families, include behavioral self-management and clinical management goals, are routinely recorded, and guide care at every subsequent point of service
**Shared PC and BH treatment plans**	1	2	3	4

	Are not available to practice teams for pre-visit planning or patient outreach	Are available to practice teams but are not routinely used for pre-visit planning or patient outreach	Are available to practice teams and routinely used for pre-visit planning or patient outreach, but only for a limited number of behavioral health disorders and risk states	Are available to practice teams and routinely used for pre-visit planning and patient outreach, across a comprehensive set of behavioral health disorders and risk states

**EHR or separate registries for behavioral health treatment needs**	1	2	3	4

	Is not provided systematically	Is limited to providing patients a list of identified community resources in an accessible format	Is accomplished through a designated staff person or resource responsible for connecting patients with community resources	Is accomplished through active coordination between the health system, community service agencies, and patients and accomplished by a designated staff person
**Linking patients to supportive community-based resources for patients with behavioral health care needs**	1	2	3	4

	Generally does not occur because the information is not available to the primary care team	Occurs only if a behavioral health provider alerts the primary care practice	Occurs because the primary care practice makes proactive efforts to identify patients with behavioral health needs	Is done routinely because the primary care practice has arrangements in place with behavioral health providers to both track these patients and ensure that follow-up is completed as indicated
**Follow up for patients with behavioral health needs**	1	2	3	4

Resources for behavioral health care needs

	Are not readily available in the practice	Are occasionally available but are limited in scope (due to some	Are generally available and usually at the level needed	Are all fully available in the practice at all times

		limitations in either staff, resources, or time)		
**Staff, resources, and time for integrated behavioral health care**	1	2	3	4

2014 CPC Behavioral Health Integration Survey

The following questions are targeted at specific behavioral health conditions.

Screening

	The practice does not screen for this disorder	The practice screens for this disorder once per year	The practice screens for this disorder when clinically indicated	The practice screens for this disorder at predetermined intervals and when clinically indicated
**Depression / mood disorders**	1	2	3	4

	The practice does not screen for this disorder	The practice screens for this disorder once per year	The practice screens for this disorder when clinically indicated	The practice screens for this disorder at predetermined intervals and when clinically indicated
**Anxiety**	1	2	3	4

	The practice does not screen for this disorder	The practice screens for this disorder once per year	The practice screens for this disorder when clinically indicated	The practice screens for this disorder at predetermined intervals and when clinically indicated
**Pain**	1	2	3	4

	The practice does not screen	The practice screens for this	The practice screens for this	The practice screens for this

	for this disorder	disorder once per year	disorder when clinically indicated	disorder at predetermined intervals and when clinically indicated
**Alcohol use disorder**	1	2	3	4

	The practice does not screen for this disorder	The practice screens for this disorder once per year	The practice screens for this disorder when clinically indicated	The practice screens for this disorder at predetermined intervals and when clinically indicated
**Cognitive function**	1	2	3	4

Outcomes

	The practice does not regularly provide care for patients with this condition	The practice treats this condition but does not have any specific treatment target	The practice treats this condition and has a treatment target	The practice treats this condition with a target, monitors regularly for treatment response, and adjusts treatment when clinically indicated, including when needed to manage side effects and complications
**Depression / mood disorders**	1	2	3	4

	The practice does not regularly provide care for patients with this condition	The practice treats this condition but does not have any specific treatment	The practice treats this treats this condition and target, monitors has a treatment target	The practice condition with a regularly for treatment

		target		response, and adjusts treatment when clinically indicated, including when needed to manage side effects and complications
**Anxiety**	1	2	3	4

	The practice does not regularly provide care for patients with this condition	The practice treats this condition but does not have any specific treatment target	The practice treats this condition and has a treatment target	The practice treats this condition with a target, monitors regularly for treatment response, and adjusts treatment when clinically indicated, including when needed to manage side effects and complications
**Pain**	1	2	3	4

	The practice does not regularly provide care for patients with this condition	The practice treats this condition but does not have any specific treatment target	The practice treats this condition and has a treatment target	The practice treats this condition with a target, monitors regularly for treatment response, and adjusts treatment when clinically indicated, including when needed to manage side

				effects and complications
**Alcohol use disorder**	1	2	3	4

	The practice does not regularly provide care for patients with this condition	The practice treats this condition but does not have any specific treatment target	The practice treats this condition and has a treatment target	The practice treats this condition with a target, monitors regularly for treatment response, and adjusts treatment when clinically indicated, including when needed to manage side effects and complications
**Cognitive function**	1	2	3	4
