# Supplementary material for: Behavioral Health and the Comprehensive Primary Care (CPC) Initiative: findings from the 2014 CPC behavioral health survey
Source: BMC Health Serv Res. 2017 Aug 29;17:612. doi: 10.1186/s12913-017-2562-z (PMC5576039; doi:10.1186/s12913-017-2562-z)
Supplement: Supplementary file 2 — 2014 Behavioral Health Integration Survey item level responses. Comprehensive Primary Care application data and the Behavioral Health Integration survey data. (PDF 72 kb) [file 12913_2017_2562_MOESM2_ESM.pdf]

ADDITIONAL FILE 2: **2014 Behavioral Health Integration Survey item level responses**

**Question**	**Answer options**	**Percentage of practices endorsing each response choice**	**Question mean (SD)**
**Type of spatial arrangement employed**	Entirely separate space	41	2.39 (1.24)
	Mostly separate space: PCPs and BHPs spend little time with each other practicing in the same clinic. Patient has to see providers in at least two buildings.	3	
	Co-located space: Behavioral health and medical clinicians in different parts of the same building, spending some but not all their time in same medical clinic space. Patient typically has to move from primary care to behavioral health space	32	
	Fully shared space: Behavioral health and medical clinicians share the same provider rooms, spending all or most of their time seeing patients in that shared space. Typically, the clinicians see the patient in the same exam room.	24	
**Training: PCPs**	Do not address behavioral health care issues	4	3.16 (0.87)
	Play a limited role in providing behavioral health care	20	
	Have been trained in principles of behavioral health care but largely rely on others to address these issues	34	
	Have been trained in principles of behavioral health care and feel comfortable handling the majority of routine behavioral health needs	43	
**Training: Other clinical staff**	Do not address behavioral health care issues	11	2.28 (0.75)
	Play a limited role in providing behavioral health care	58	
	Have been trained in principles of behavioral health care but largely rely on others to address these issues	24	

	Have been trained in principles of behavioral health care and feel comfortable handling the majority of routine behavioral health needs as allowed by their license	7	
**Training: Non-clinical staff**	Do not address behavioral health care issues	52	1.66 (0.84)
	Are primarily tasked with managing patient flow and triage regarding behavioral health needs	34	
	Provide some services such as assessment or screening for behavioral health conditions	9	
	Perform key service roles that match their abilities and position	5	
**Access: Appointments with PCPs to address behavioral health needs**	Are not available at the practice	3	3.08 (0.77)
	Can be scheduled by the practice with some flexibility in scheduling different visit lengths	17	
	Provide flexibility and include capacity for same day visits	49	
	Are flexible and can accommodate customized visit lengths, same day visits, schedule follow-up, and multiple provider visits	31	
**Access: Appointments with BHPs to address behavioral health needs**	Are not available at this practice	42	2.21(1.22)
	Can be scheduled by the practice with some flexibility in scheduling different visit lengths	20	
	Provide flexibility and include capacity for same day visits	14	
	Are flexible and can accommodate customized visit lengths, same day visits, schedule follow-up, and multiple provider visits	24	
**Communication and coordination: PCPs and BHPs**	Do not regularly communicate; BHPs are not part of the practice	22	2.43 (1.08)
	Engage in periodic information exchanges with minimally shared care plans or workflows	38	

	Engage in regular communication and coordination, usually via separate systems and workflows, but with care plans coordinated to a significant extent	16	
	Have regular communication facilitated and/or clinical workflows that ensure effective communication and coordination	24	
**Communication and coordination: Case review**	PCPs and BHPs do not meet regularly to review cases	63	1.61 (0.94)
	PCPS and BHPS meet periodically to conduct caseload review on patients who are not improving	22	
	PCPS and BHPs meet routinely to conduct caseload review on patients who are not improving	7	
	PCPs and BHPs meet routinely to conduct caseload review on patients who are not improving; BHPs provide specific recommendations for treatment changes or referrals, and provide psychiatric assessment when indicated	8	
**Treatment planning: Shared PC and BH treatment plans**	Are not routinely developed or recorded	25	2.24 (0.99)
	Are developed and recorded but reflect PCP and BHPs separate priorities only	40	
	Are developed collaboratively with PCPs and BHPs and include behavioral self-management and clinical goals, but they are not routinely used to guide subsequent care	21	
	Are developed collaboratively with PCPS and BHPS as well as patients and families, include behavioral self-management and clinical management goals, are routinely recorded, and guide care at every subsequent point of service	14	
**Treatment planning: EHR or separate registries for behavioral health treatment needs**	Are not available to practice teams for pre-visit planning or patient outreach	39	2.09 (1.05)
	Are available to practice teams but are not routinely used for pre-visit planning or patient outreach	26	

	Are available to practice teams and routinely used for pre-visit planning or patient outreach, but only for a limited number of behavioral health disorders and risk states	24	
	Are available to practice teams and routinely used for pre-visit planning and patient outreach, across a comprehensive set of behavioral health disorders and risk states	12	
**Treatment planning: Linking patients to supportive community-based resources for patients with behavioral health care needs**	Is not provided systematically	8	2.75 (0.87)
	Is limited to providing patients a list of identified community resources in an accessible format	29	
	Is accomplished through a designated staff person or resource responsible for connecting patients with community resources	42	
	Is accomplished through active coordination between the health system, community service agencies, and patients and accomplished by a designated staff person	21	
**Treatment planning: Follow up for patients with behavioral health needs**	Generally does not occur because the information is not available to the primary care team	11	2.65 (0.87)
	Occurs only if a behavioral health provider alerts the primary care practice	30	
	Occurs because the primary care practice makes proactive efforts to identify patients with behavioral health needs	44	
	Is done routinely because the primary care practice has arrangements in place with behavioral health providers to both track these patients and ensure that follow-up is completed as indicated	16	
**Resources for behavioral health care needs: Staff, resources and time for integrated behavioral health care**	Are not readily available at this practice	19	2.38 (0.95)
	Are occasionally available but are limited in scope (due to some limitations in either staff, resources, or time)	38	

	Are generally available and usually at the level needed	29	
	Are all fully available in the practice at all times	14	
**Screening: Depression/mood disorders**	The practice does not screen for this disorder	3	3.47 (0.75)
	The practice screens for this disorder once per year	6	
	The practice screens for this disorder when clinically indicated	31	
	The practice screens for this disorder at predetermined intervals and when clinically indicated	60	
**Outcomes: Depression/mood disorders**	The practice does not regularly provide care for patients with this condition	1	3.01 (0.91)
	The practice treats this condition but does not have any specific treatment target	37	
	The practice treats this condition and has a treatment target	22	
	The practice treats this condition with a target, monitors regularly for treatment response, and adjusts treatment when clinically indicated, including when needed to manage side effects and complications	40	
**Screening: Anxiety**	The practice does not screen for this disorder	21	2.84 (1.05)
	The practice screens for this disorder once per year	2	
	The practice screens for this disorder when clinically indicated	49	
	The practice screens for this disorder at predetermined intervals and when clinically indicated	28	
**Outcomes: Anxiety**	The practice does not regularly provide care for patients with this condition	5	2.82 (0.97)
	The practice treats this condition but does not have any specific treatment target	42	

	The practice treats this condition and has a treatment target	19	
	The practice treats this condition with a target, monitors regularly for treatment response, and adjusts treatment when clinically indicated, including when needed to manage side effects and complications	34	
**Screening: Pain**	The practice does not screen for this disorder	17	2.89 (1.01)
	The practice screens for this disorder once per year	4	
	The practice screens for this disorder when clinically indicated	50	
	The practice screens for this disorder at predetermined intervals and when clinically indicated	29	
**Outcomes: Pain**	The practice does not regularly provide care for patients with this condition	9	2.89 (1.03)
	The practice treats this condition but does not have any specific treatment target	31	
	The practice treats this condition and has a treatment target	21	
	The practice treats this condition with a target, monitors regularly for treatment response, and adjusts treatment when clinically indicated, including when needed to manage side effects and complications	39	
**Screening: Alcohol use disorder**	The practice does not screen for this disorder	13	3.09 (1.04)
	The practice screens for this disorder once per year	11	
	The practice screens for this disorder when clinically indicated	31	
	The practice screens for this disorder at predetermined intervals and when clinically indicated	45	

**Outcomes: Alcohol use disorder**	The practice does not regularly provide care for patients with this condition	29	2.18 (1.08)
	The practice treats this condition but does not have any specific treatment target	39	
	The practice treats this condition and has a treatment target	16	
	The practice treats this condition with a target, monitors regularly for treatment response, and adjusts treatment when clinically indicated, including when needed to manage side effects and complications	16	
**Screening: Cognitive function**	The practice does not screen for this disorder	14	2.89 (0.93)
	The practice screens for this disorder once per year	6	
	The practice screens for this disorder when clinically indicated	56	
	The practice screens for this disorder at predetermined intervals and when clinically indicated	24	
**Outcomes: Cognitive function**	The practice does not regularly provide care for patients with this condition	14	2.49 (0.99)
	The practice treats this condition but does not have any specific treatment target	46	
	The practice treats this condition and has a treatment target	18	
	The practice treats this condition with a target, monitors regularly for treatment response, and adjusts treatment when clinically indicated, including when needed to manage side effects and complications	22	
